# The Upsides and Downsides of the Dark Side: A Longitudinal Study Into the Role of Prosocial and Antisocial Strategies in Close Friendship Formation

**DOI:** 10.3389/fpsyg.2019.00114

**Published:** 2019-02-19

**Authors:** Joseph Ciarrochi, Baljinder K. Sahdra, Patricia H. Hawley, Emma K. Devine

**Affiliations:** ^1^Institute of Positive Psychology and Education, Australian Catholic University, Sydney, NSW, Australia; ^2^Texas Tech University, College of Education, Lubbock, TX, United States

**Keywords:** sex differences, resource control theory, well-being, self-concept and self esteem, empathy

## Abstract

Resource control theory (RCT) posits that both antisocial and prosocial behaviors combine in unique ways to control resources such as friendships. We assessed students (*N* = 2,803; 49.7% male) yearly from junior (grades 8–10) to senior high school (11–12) on antisocial (A) and prosocial (P) behavior, peer nominated friendship, and well-being. Non-parametric cluster analyses of the joint trajectories of A and P identified four stable profiles: *non-strategic* (moderately low A and P), *bi-strategic* (moderately high on A and P), *prosocial* (moderately low A and moderately high on P), and *antisocial* (moderately low on P, and very high on A). There were clear benefits to youth using bi-strategic strategies in junior high: they attracted relatively high levels of opposite sex friendship nominations. However, this benefit disappeared in senior high. There were also clear costs: *bi-strategic* youth experienced relatively low well-being, and this effect was significantly more pronounced for females than males. *Prosocial* youth were the only ones who maintained both high friendship numbers and high well-being throughout high school. We discuss the cost/benefit trade-offs of different resource control strategies.

## Introduction

What is the key to building strong social networks? Many researchers offer an intuitively appealing answer: we need to be prosocial. Cooperate. Support others. Take their perspective. Give. For example, Ciarrochi et al. (2016a) argue that empathy helps youth communicate, resolve conflict, and engage in prosocial behavior, all of which helps them build close friendships. Wilson et al. (2014) argue that people who are skillful at cooperating are able to outperform and survive those who fail to cooperate. Grant (2014) argues that “givers,” rather than takers, are able to build strong social networks that help them dominate the top of the success ladder. Perhaps being prosocial really is the best path to success. But this conclusion leaves us with an important question. Why are so many antisocial people also socially successful?

People can benefit from using antisocial or coercive strategies, which in this paper refers to aggression, rule breaking, and deception. Aggressive youth can be viewed by their peers as leaders (Waasdorp et al., 2013, and as fun, charming, and prestigious (Hawley, 2003; Hawley et al., 2007). Bullies are more likely than others to be popular (Wegge et al., 2016; Duffy et al., 2017; Pouwels et al., 2018). Youth who are above average in aggression have the highest social competence (Bukowski, 2003). Findings like these lead us to conclude that both antisocial and prosocial behaviors are linked to socially effective behavior (Hawley, 1999; Kashdan and Biswas-Diener, 2014). Thus, we need to focus our question not on *if* prosocial and antisocial behaviors are effective, but *when* they are effective and for what.

The present paper sought to evaluate when the prosocial and antisocial behaviors of youth are associated with positive social consequences, which, in this study, were operationalized as number of peers that nominated a young person as a close friend. We utilized archival data from a 5-year, longitudinal cohort study that spanned grades 8–12 and repeatedly assessed cognitive and affective empathy (prosocial indices), and aggression and rule breaking (antisocial indices). Whilst our focus was on friendship outcomes, we also examined psychological outcomes (mental health and self-esteem). This allowed us to assess the possibility that antisocial and prosocial strategies can have a social benefit but a psychological cost, and *vice versa*. We hypothesized that the effect of social strategies on youth social connection and well-being would depend on three factors: the young person’s configuration of antisocial and prosocial behavior usage (e.g., low in both, versus high in at least one), the type of friend (same versus opposite sex), and the developmental stage of the youth (junior versus senior high).

### Individual **D**ifferences in **S**ocial **C**ontrol **S**trategy

#### Theories of Aggressive and Empathic Behavior

For the purposes of this review we will focus on one subset of antisocial behavior, namely aggression, or behavior intended to harm another person. A large number of theories have offered different explanations for aggression, including the notion that, under specific circumstances, aggression is the result of frustration and aversive affect (Dollard et al., 1939; Berkowitz, 1989), social learning (Bandura, 1986), and hostile attribution bias (Crick and Dodge, 1994). The General Aggression Model (GAM) seeks to bring all of these theories together within one unifying model (Allen et al., 2018). This theory assumes that a person’s internal state is due to relatively stable factors (i.e., individual differences in biology) as well as interactions between the person and the situation. When people experience an internal state such as frustration, they engage in appraisal and a decision process that leads to either thoughtful or impulsive action, both of which may be aggressive.

Like aggression theories, there are number of empathy theories focused on different aspects of the empathy process. Some theories focus on biological factors (e.g., “mirror neurons” neural circuits; Keysers and Gazzola, 2009), whilst others focus on behavior, such as automatic mimicry and feedback (Hatfield et al., 1994; Hoffman, 2001), learning processes (Hoffman, 2001), verbal perspective taking (McHugh et al., 2012), appraisals (Wondra and Ellsworth, 2015), and motivation to avoid distress and costs and experience positive affect and rewards (Zaki, 2014).

These theories form an important backdrop to the present study in that they seek to explain the individual differences and contexts that elicit aggressive and empathic behaviors. The present study focuses on the function, or consequences of these behaviors for adolescent friendships.

#### Resource Control Theory

Hawley’s resource control theory (RCT) provides one functional and evolutionary account of the role of prosociality and antisociality in human psychological and social functioning. Namely, resources of various types (material, informational, and social) are important for physical and cognitive growth, and well-being. Accordingly, competition—both overt and covert—is an essential part of human nature, with strategies to be used alone or in combination (Hawley, 1999). Friendships can be viewed as a limited resource that peers seek to access, cultivate, and protect. Friendships are the source of access to material goods, social status, social need satisfaction, information, support for goals, and defense against bullies (Hawley, 2003; Hawley et al., 2009). Youth compete for friendships using both coercion (aggression and deception toward, for example, third party threats) and prosocial behavior (empathic behavior, helping, reciprocation and cooperation).

Hawley and Little (2002) have hypothesized different “resource control types,” or subgroups of people that emphasize the use of either coercive or prosocial strategies. Resource control subgroups can be identified in a number of ways, but one common procedure is to administer a measure of coercive and prosocial behavior, and then divide the distribution of coercive and prosocial people into thirds (33%, 66%). This approach has led to the identification of five resource control subgroups: *Prosocial* controllers (high on prosocial, low or average on antisocial strategies), coercive controllers (high antisocial, and average or low prosocial), *bi-strategic* controllers (high on both strategies), non-controllers (low on both strategies), or typical controllers (average on both strategies).

Both coercion and prosociality has been theorized as socially effective (Hawley, 1999), but using both strategies in tandem has been hypothesized to be especially effective in garnering social resources (Hawley and Little, 2002; Hawley, 2003, 2014; Hawley et al., 2007; Wursterm and Xie, 2014). Individuals with capabilities in both domains would have more flexibility in pursuing their social goals, because they can both utilize the social group to access resources in some contexts and bypass or confront the group in others, in calculated ways (Hawley et al., 2009). As such, they are expected to have positive characteristics in common with *prosocial* controllers (social competencies, social attractiveness) as well as the less attractive characteristics of coercive controllers (hostility, unethicality). Though seemingly paradoxical on its face, *bi-strategics* are expected to utilize skills associated with prosocial behavior (e.g., perspective taking) to effectively implement aggression or more subtle forms of control.

Past research supports the validity of this type of classification, in that resource control types relate in theoretically coherent ways to resource acquisition and goal attainment (e.g., popularity, material gain, preferential treatment from authority), personality traits and motivations (Hawley, 2003; Hawley et al., 2007, 2009; Wursterm and Xie, 2014). For example, *prosocial* controllers (Grades 3–6) tend to have intrinsic friendship motivation (e.g., motivated by the joy friendships bring), whereas coercive controllers tend to have extrinsic motivation (e.g., power and status). As would be expected of children who report both strategies, *bi-strategic* controllers were found to be high in both kinds of motivation and, accordingly, report the highest level of peer influence (Hawley and Little, 2002).

Similarly, research involving elementary school children suggests that *bi-strategics* are often the most effective at some aspects of resource control (e.g., achieving popularity, social dominance; Hawley et al., 2008; Chen and Chang, 2012; Wursterm and Xie, 2014). In a study focused on adolescence (Grades 7–10), *bi-strategic* adolescents were rated highest on intimacy (as one might expect when one is intrinsically motivated) but were also rated highest in conflict (presumably a consequence of high need for control; Hawley et al., 2007). While *prosocial* controllers received the highest quantity of best friend nominations, *bi-strategic* youth received the second highest. Coercive controllers and non-controllers tied for fewest friendship nominations. This latter point illustrates, poignantly, the flip side to the status coin: using no strategies at all leads to utter lack of success in the peer group. Friendships clearly are not merely won by lack of aggression. Agency is also required. It is also worth noting that past research focused on same sex friendship nominations and did not systematically explore same and opposite sex friendships, as we do in the present study.

The above studies formed subgroups based on arbitrary cut-off scores. It is also possible to utilize statistical methods to derive cut-offs, such as Latent class analysis. In one such study, which focused on youth from Chile (Grades 4–6), no strictly *bi-strategic* profile was found. Instead, these authors identified three subgroups: normative-non-aggressive (low aggressive, average on prosocial variables); high prosocial-low aggressive; and high-aggressive-high popular. Thus, Hawley’s (2012) originally proposed resource control subtypes may, in part, be susceptible to cultural factors (Berger et al., 2015).

Similar to Berger et al. (2015), the present study utilized a data-driven, cluster-analytic approach. In principle, many different social behavior profiles may occur in the population, which differ not only in the extent that they deviate from mean levels of prosociality and antisociality, but also the size of the deviation. For example, we could identify a profile that is extremely high in antisocial behavior and prosociality, and another group that is only moderately high in antisociality, and extremely low on prosociality. Alternatively, we may fail to identify one of the groups defined using human cut-offs in past research. Our present approach allows us to explore this possibility. Will we replicate Hawley’s five profiles using a data-driven, longitudinal cluster analysis approach (±1 SD) used in past research (i.e., will we identify the same profiles with approximately the same cut points; **Research question 1**)? In addition, the longitudinal nature of our data set allowed us to address two additional questions. Does the composition of resource control type change across high school (e.g., does the antisocial group become increasingly antisocial; **Research question 2**). Do the effects of resource control type change across high school (e.g., do antisocial youth receive fewer nominations in senior high school than junior high school; **Research question 3**).

### Social Control Strategy and Opposite Sex Friendships

Same sex friendships predominate during the pre-adolescent period (Bukowski et al., 1993; Maccoby, 1998). However, by early adolescence, youth start spending more time with other sex peers, and start forming other sex friendships and best friendships (Darling et al., 1999; McDougall and Hymel, 2007). These types of friendships are distinct from romantic relationships and same sex friendships (McDougall and Hymel, 2007). Eighth grade boys and girls rate mixed sex contexts as more enjoyable than same sex contexts (Darling et al., 1999).

How can a young person’s antisocial and prosocial tendencies attract opposite sex friendships? Ciarrochi et al. (2016a) have argued that having social strategies, such as asking people how they are feeling (empathy), increase your chance of being “detected” by opposite sex peers, but should have less role in detection amongst same sex peers. This is because same sex peers typically have had more extensive experience with each other than they tend to have with opposite sex peers (Bukowski et al., 1998). Research suggests that increased experience is associated with increased detection of trait cues (Funder, 1995). Accordingly, we hypothesize that social strategies should increase opposite sex visibility more than they increase same sex visibility, given same sex peers have other ways to be visible (e.g., shared activities). *Non-strategic* males and females should be virtually invisible to the opposite sex, and therefore unlikely to be nominated as a close friend. One of the interesting implications of this hypothesis is that although *non-strategic* youth are less negative than the *antisocial* group, they will nevertheless have fewer friends than the *antisocial* group. Based on RCT, we predict that *non-strategic* youth will receive the fewest nominations of any group for both same and opposite sex relationships (**Hypothesis 1a**). We also predict that this effect will be strongest in opposite sex relationships (**Hypothesis 1b**).

The social/perspective taking skills of *bi-strategic* and *prosocial* youth are expected to give both groups an advantage in attracting friends. However, the antisocial component of *bi-strategics* is expected to have different costs and benefits in same and opposite sex relationships. In opposite sex relationships, the *bi-strategics* may be able to use aggression in a skillful way and their behavior may be viewed as an indication of social dominance, confidence, and charisma. This should lead to a relatively high number of opposite sex friendship nominations.

In contrast, the aggressive behavior of bi-strategic youth is expected to be relatively aversive and status threatening to same sex peers, who belong to the same social hierarchy. That is, we expect a female’s status to be more likely to be threatened by socially dominant females than socially dominant male’s status, and *vice versa*. Consistent with this view, Ciarrochi and Heaven (2009) found that a trait associated with social dominance, extraversion, was less strongly associated with same sex liking nominations than opposite sex nominations. Similarly, Kerestes and Milanovic (2007) found that aggressive children of both genders tended to be more rejected by their same sex peers than opposite sex peers. Based on RCT and this past research, we predict that *bi-strategic* youth will receive more same sex friendship nominations than all groups except *prosocial* (**Hypothesis 2**). In addition, Hypothesis 3, like Hypothesis 1, predicts that those with more negative qualities (“*bi-strategics*”) will receive more opposite sex nominations than those with less negative qualities (“*Prosocial*”) (**Hypothesis 3**).

### Social Versus Psychological Well-Being

We have argued above that being *bi-strategic* has social benefits, especially in attracting opposite sex nominations. Here, we will argue that the same profile could have psychological costs. Specifically, coercive (*bi-strategic* and *antisocial*) youth may struggle with forming genuine relationships, because they are afraid of intimacy, and are more likely to engage in intimacy interfering behaviors such as using people as a means to an end and bullying (Hawley et al., 2009; Zeigler-Hill et al., 2014). In addition, coercive youth tend to experience particularly low relationship confidence (Hawley et al., 2009).

Substantial theory and related research suggest that genuine social connection is a basic psychological need that is universal to all humans, even to those who say they don’t value relationships (Ryan and Deci, 2017). Thus, we predict that coercive youth are less likely than their counterparts to have their social needs met and more likely to have relatively low self-esteem and mental health (**Hypothesis 4**).

## Study

The present study assessed a large cohort of students yearly from junior high (Grades 8–10) and high school (Grades 11–12) on levels of antisocial behavior, empathy, peer nominated friendship, and self-reported well-being. Empathy and antisocial behavior were measured only in Grades 8–11. The outcome variables—peer nominated friendship, mental health, and self-esteem—were measured in Grades 8 through 12.

### Sample and Procedure

The sample consisted of a cohort of students from 16 secondary schools within the Cairns (QLD) and Illawarra (NSW) Catholic Dioceses of Australia. All schools within the Dioceses participated. This sample was part of a larger project (name blinded for review), in which participants completed a battery of questionnaires. Paper-and-pencil questionnaires were administered using a similar procedure in all schools. Ethics approval was obtained for the (Australian Character Study) study from the (Australian Catholic University) Human Research Ethics Committee (HE10/158) before data collection. Written informed consent was obtained from both the participants and the parents of the participants.

Students completed the empathy and antisocial measures in Grades 8–11, but the measures were not included in Grade 12 due to time limitations set by the schools. Mental health, self-esteem, and friendship nominations were administered in Grades 8 through 12. All youth that completed empathy and antisocial behavior in at least one wave of data were included in the study. In total, 2,803 students (49.7% males) completed questionnaires (see the analysis section for additional details on missing data). The questionnaires were administered in October and November of each year, which is toward the end of the school year in Australia. The sample varied somewhat from year to year due to schools being the primary sampling unit. Thus the sample changed slightly as youth left the school, joined the school, or were absent on the day of testing. The students completed assessments in Grade 8 (*M*_age_ = 13.7, SD_age_ = 0.45; *n* = 2,063), Grade 9 (*M*_age_ = 14.7, SD_age_ = 0.46; *n* = 2,081), Grade 10 (*M*_age_ = 15.7, SD_age_ = 0.44; *n* = 2,019), Grade 11 (*M*_age_ = 16.6, SD_age_ = 0.46; *n* = 1,735), and Grade 12 (*M*_age_ = 17.7, SD_age_ = 0.40; *n* = 1,591). Refusal to participate was negligible. Seven-hundred and ninety students completed all time waves, 1,997 completed at least three time waves, and 376 completed only one time wave. The demographic makeup of this sample broadly reflects that of the Australian population in terms of ethnicity, employment, and religious belief (Author calculation based on Australian Bureau of Statistics, 2012). The Australian Government provides a school socioeconomic index in which the average across Australia is 1,000^1^. The schools in this sample had a similar average score of 1,026 (SD = 43).

### Measures

#### Self-Esteem

Global trait self-esteem was measured using the 10-item Rosenberg self-Esteem scale (RSE; Rosenberg, 1965). Participants were asked to indicate their agreement with statements such as, “Generally I feel satisfied with myself” and “I think that I am a failure.” A binary forced response scale (“yes” or “no”) was utilized. This scale has been validated in previous youth research (Marshal et al., 2013), and showed good internal consistency in the present sample (α_8_ = 0.85; α_9_ = 0.86; α_10_ = 0.88; α_11_ = 0.88; α_12_ = 0.86).

#### Mental Health

General ill-health was measured using the General Health Questionnaire, which is a highly used, reliable, and valid measure of mental health (Golderberg and Hillier, 1979) that has been successfully used with adolescents (Tait et al., 2003; Ciarrochi et al., 2016b). Participants were provided with the sentence stem, “Have you recently…” and then with 12 response-items including, “been feeling unhappy or depressed” and “felt you couldn’t overcome your difficulties.” Ratings were made on a four-point scale, with labels such as “*not at all*” to “*much more than usual*.” Higher scores are indicative of greater psychological distress. In the present sample, the measures showed strong internal consistency (α_8_ = 0.89; α_9_ = 0.89; α_10_ = 0.90; α_11_ = 0.91; α_12_ = 0.90).

#### Friendship Nominations

There are a number of valid ways to collect peer ratings, including the round-robin system and peer nomination systems, and procedures that present participants with classmate names and those that ask participants to recall names (Cillessen, 2009). In the present article, we asked people to nominate their closest friends. We considered it unlikely that participants would forget the names of their closest friends, and so we utilized the relatively simple and quick peer recall system. Our approach made it easy to collect substantial amounts of data from multiple schools.

We asked students to nominate up to five of their closest male and five closest female friends in the same year group at their school (Rowsell et al., 2014; Parker et al., 2015). This approach is a modification of procedures used for several decades to understand childhood and teenage friendships (Coie et al., 1982).

#### Resource Control

There are a substantial number of ways to measure prosocial and coercive behavior. In principle, there are three dimensions of any social behavior: the strategy type (e.g., coercive or prosocial), the function or goal (to gain information, to build closeness and satisfy social needs), and the effectiveness of the strategy (did it work in meeting the intended goal?). No single measure captures every single aspect of these dimensions. In the present study, based on archival data, we had measures that focus exclusively on strategy type (e.g., does a youth take perspective and share the feelings of others, is a youth aggressive and rule breaking)?

##### Empathy

We used the Basic Empathy Scale (BES; Jolliffe and Farrington, 2006a) to assess both cognitive and affective empathy. Cognitive empathy refers to the capacity to comprehend the emotions of another and is measured with items like “I find it hard to know when my friends are frightened” (reverse-scored), “When someone is feeling down I can usually understand how they feel,” and “I can often understand how people are feeling even before they tell me.” Affective empathy refers to the capacity to experience the emotions of another and is measured with items like “I get caught up in other people’s feelings easily,” “I often get swept up in my friend’s feelings” and “After being with a friend who is sad about something, I usually feel sad.” Cognitive and affective empathy are intercorrelated yet clearly distinguishable (Jolliffe and Farrington, 2006a). The BES relates in expected ways to other empathy measures, to personality measures, to low levels of antisocial behavior, to high levels of pro-social behavior, and to differences in brain activity (Jolliffe and Farrington, 2006a,b; Albiero et al., 2009; Sebastian et al., 2012; Sahdra et al., 2015). The BES subscales showed good internal consistency in the present sample (affective empathy α_8_ = 0.77; α_9_ = 0.79; α_10_ = 0.81; α_11_ = 0.83; Cognitive Empathy: α_8_ = 0.76; α_9_ = 0.79; α_10_ = 0.80; α_11_ = 0.84).

##### Antisocial behavior

The 31 problem items of the Achenbach (1991) Youth Self-Report inventory has participants rate the extent that statements are true of them, ranging from 0 *not true*, 1 *somewhat or sometimes true*, and 2 *very true or often true*. The Rule-breaking subscale of the YSR consists of 15 items and examines such behavior tendencies as lying, stealing, and breaking rules. The Aggression subscale of the YSR consists of 16 items and includes behaviors such as arguing, fighting with other children, destroying things, and bullying others. This scale is widely used and validated (Ivanova et al., 2007; Semel, 2017). It showed good internal consistency in the present sample (rule breaking α_8_ = 0.84; α_9_ = 0.88; α_10_ = 0.85; α_11_ = 0.83; Aggression: α_8_ = 0.88; α_9_ = 0.90; α_10_ = 0.87; α_11_ = 0.86).

### Analyses Plan

#### Missing Data

There was little in the way of unit non-response within each wave. Yet, as noted above, the school was the primary sampling unit and, thus, attrition was moderate and generally represented attrition from the school. To deal with missing values, we used the copy mean imputation, which is a two-step procedure in which linear interpolation based on the existing data is first used to impute a value and in a second step the value is updated such that it is shrunk toward the average trajectory (Genolini and Jacqmin-Gadda, 2013). This method is designed specifically for longitudinal trajectory data and has been shown to perform well under MAR, MCAR, and even NMAR missing data mechanisms (Genolini and Jacqmin-Gadda, 2013).

#### Cluster and Growth Analyses

The main growth analyses utilized the R package KmL3d, which employs a non-parametric algorithm for clustering joint trajectories (Genolini et al., 2013, 2015). We utilized a non-parametric algorithm to capture potential discontinuities in the rate of change of trajectories, particularly around Grade 10, the transition from junior to senior high school. The optimal number of developmental profiles was chosen by selecting the best fitting model based primarily on the Calinski and Harabatz (CH) criterion. We also considered the Ray and Turi (RT), Davies and Bouldin (DB), and BIC criterion [see Genolini et al. (2013)]. Ultimately, the number of profiles should be determined by a combination of factors in addition to fit indices, including theoretical justifications and interpretability (e.g., our expectation of a stable profile) (Ciarrochi et al., 2017; Morin et al., 2017).

KmL3d provides class membership probabilities for each individual. Instead of using an “all-or-none” approach of assigning class membership to participants based on the highest probability for one of the profiles, we employed a more sensitive, graded approach. Using each individual’s estimated probability of membership for each class as sampling probabilities, we sampled each individuals class membership 25 times, creating 25 datasets that are akin to multiple imputations (Sahdra et al., 2016). All statistical tests were subsequently run using these imputations with results combined using Rubin (2004) rules. This allowed us to account for uncertainty in the latent class membership. We also utilized both *k*-means and *k*-medians clustering to ensure that results replicated across analyses types. Both analyses identified the same basic profiles. The results of the *k*-medians analyses are presented in Supplementary Table S7. The *k*-means analysis is presented in detail below.

#### Multilevel Negative Binomial Analyses

The outcome variable, friendship nomination, was count data. We utilized negative binomial regressions because the outcome, friendship nominations, was count data. The data constituted a hierarchically nested data structure in that peer nomination counts are nested within individuals and individuals are nested within schools. Relationships between peer nominations and profile were analyzed using multilevel random coefficient models (MRCMs) as implemented in the R program lme4 (Bates et al., 2014). The nested multilevel models in the lme4 framework use all available information for each person. Friendship counts were predicted by profile membership, gender of the nominator, gender of the nominee, and stage in high school, all interactions involving these variables, and grade level of student. Stage in high school had two levels, junior high (Grades 8–10) and senior high (Grades 11 and 12).

## Results

### Preliminary Analyses

The descriptive statistics for the study variables for each gender are presented in Supplementary Table S1. Here, we summarize the most important trends observed across the 5 years of the study. The mean level of aggression (*M*_m_ = 0.40–0.46, SD_m_ = 0.35–0.41; *M*_f_ = 0.38–0.44, SD_f_ = 0.32–0.38) and rule breaking (*M*_m_ = 0.37–46, SD_m_ = 0.30–0.39; *M*_f_ = 0.29–0.37, SD_f_ = 0.28–0.36) indicate that, on average, students viewed the antisocial statements as not true of them (0) or (1) only somewhat or sometimes true. There was little or no difference between males and females in reported aggression (Cohen’s *d* < 0.06), but males tended to break rules more than females (*d* < 0.30). The following are the means for the other study variables, with effect sizes (Cohen’s *d*) for gender contrasts: affective empathy (*M*_m_ = 2.83–3.17, SD_m_ = 0.51–0.65; *M*_f_ = 3.4–3.73, SD_f_ = 0.51–0.63; *d* = 0.88–1.12), cognitive empathy (*M*_m_ = 3.77–3.94, SD_m_ = 0.56–0.63; *M*_f_ = 4.06–4.19, SD_f_ = 0.52–0.55; *d* = 0.44–0.59), self-esteem (*M*_m_ = 0.72–0.76, SD_m_ = 0.23–0.26; *M*_f_ = 0.59–0.65, SD_f_ = 0.28–0.30; *d* = 0.43–0.62), mental health (*M*_m_ = 1.76–1.97, SD_m_ = 0.49–0.53; *M*_f_ = 1.95–2.25, SD_f_ = 0.54–0.61; *d* = 0.37–0.51).

We utilized Spearman correlations in all analyses, given our variables did not tend to be normally distributed (e.g., friendship counts followed a negative binomial distribution). All correlations greater than 0.08 were significant at *p* < 0.05. Across the 5 years of the study, affective and cognitive empathy tended to have small to non-significant links with aggression (*r* = 0.06 to *r* = -0.15) and small, negative links with rule breaking (*r* = -0.07 to *r* = -0.18). Antisocial behavior showed little or no link to friendship nominations amongst males and females (*r* = -0.07–0.19; average *r* = 0.01; see Supplementary Table S2). Similarly, cognitive and affective empathy had little relationship with the extent males nominated females as friends (*r* = -0.05–0.15; average *r* = 0.06) or males as friends (*r* = -0.01–0.13; average *r* = 0.07; see Supplementary Table S3). Empathy was unrelated to the extent that females nominated females as friends (*r* = 0–0.08, mean *r* = 0.04). A significant association was observed for the extent that females nominated males as friends (*r* = 0.10–0.27, mean *r* = 0.18).

We next examined the stability of our study variables. Aggression (average *r* = 0.66), rule breaking (average *r* = 0.64), and affective empathy (average *r* = 0.66) showed the highest stability from year to year, followed by cognitive empathy (average *r* = 0.53). Concerning peer rated friendships, female friendship preferences for females (average *r* = 0.62) and males (average *r* = 0.62) were moderately stable, and somewhat more stable than male friendship preferences for males (average *r* = 0.53) and females (average *r* = 0.43).

### Identifying Number of Profiles

**Figure 1:**
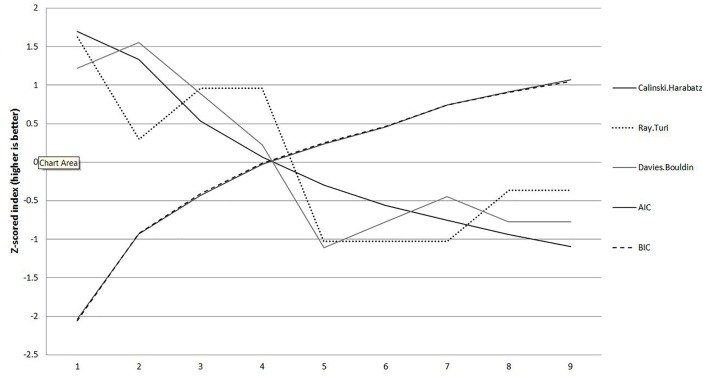
Fit indices for different profile solutions. Horizontal bar indicates four profile cut-off.

Utilizing statistical methods, rather than pre-defined splits, do we replicate Hawley’s five human derived profiles (**Research question 1**)? The fit indices for each profile are presented graphically in Figure 1, with all indices standardized such that higher scores indicate better fit. Calinski-Harabaz, Ruy-Turi, and Davies-Bouldin indices all suggest worsening fit with each added profile. There was an especially steep drop in fit after four profiles. In contrast, AIC show improving fit, leveling off at about five profiles.

We next examined the profile solutions for theoretical meaningfulness. The four profile solution is illustrated in Table 1, and profile solutions 3, 5, and 6 are presented in the Supplementary Tables S4–S6. The four profiles where labeled *non-strategic*, *prosocial*, *bi-strategic*, and *antisocial*. The *bi-strategic* group, whilst being clearly above average in antisocial behavior and rule breaking, tended to engage in fewer of these behaviors than the *antisocial* group. The *antisocial* group tended to have extremely high scores on the antisocial indices, whilst being moderately low in empathy. The *non-strategic* group were moderately low on all dimensions, whereas the *prosocial* group were moderately low on antisocial indices but moderately high on *prosocial* indices.

**Table 1:** Characteristics of the four profiles identified in non-parametric joint trajectory cluster analysis.

	*Non-strategic*			Prosocial			*Bi-strategic*			*Antisocial*		
	*N* = 992	95% CI		*N* = 992	95% CI		*N* = 501	95% CI		*N* = 295	95% CI	
	*M*	*LL*	*UL*	*M*	*LL*	*UL*	*M*	*LL*	*UL*	*M*	*LL*	*UL*
Grade 8								
Aggressive	-0.31	-0.35	-0.27	-0.5	-0.53	-0.47	0.75	0.67	0.82	1.46	1.32	1.6
Rule breaking	-0.24	-0.28	-0.21	-0.5	-0.53	-0.47	0.51	0.44	0.59	1.64	1.48	1.8
Affective emp.	-0.57	-0.62	-0.52	0.49	0.44	0.54	0.56	0.49	0.63	-0.65	-0.76	-0.53
Cognitive emp.	-0.54	-0.60	-0.49	0.54	0.49	0.59	0.36	0.29	0.43	-0.57	-0.71	-0.43
Grade 9								
Aggressive	-0.37	-0.34	-0.27	-0.54	-0.58	-0.52	0.82	0.57	0.71	1.68	1.56	1.8
Rule breaking	-0.3	-0.34	-0.27	-0.55	-0.58	-0.52	0.64	0.57	0.71	1.78	1.65	1.92
Affective emp.	-0.64	-0.69	-0.59	0.59	0.54	0.64	0.61	0.54	0.67	-0.83	-0.94	-0.73
Cognitive emp.	-0.57	-0.62	-0.51	0.6	0.56	0.64	0.38	0.32	0.45	-0.74	-0.89	-0.6
Grade 10								
Aggressive	-0.37	-0.41	-0.34	-0.51	-0.54	-0.48	0.78	0.71	0.85	1.64	1.51	1.78
Rule breaking	-0.29	-0.33	-0.26	-0.55	-0.58	-0.52	0.63	0.56	0.71	1.69	1.56	1.83
Affective emp.	-0.64	-0.69	-0.59	0.62	0.57	0.66	0.58	0.52	0.64	-0.84	-0.95	-0.73
Cognitive emp.	-0.58	-0.63	-0.53	0.61	0.57	0.65	0.39	0.33	0.46	-0.76	-0.91	-0.62
Grade 11								
Aggressive	-0.34	-0.38	-0.3	-0.51	-0.54	-0.48	0.72	0.66	0.79	1.64	1.51	1.78
Rule breaking	-0.25	-0.29	-0.21	-0.55	-0.58	-0.52	0.59	0.52	0.66	1.69	1.56	1.83
Affective emp.	-0.61	-0.66	-0.56	0.59	0.55	0.64	0.53	0.46	0.6	-0.84	-0.95	-0.73
Cognitive emp.	-0.56	-0.61	-0.5	0.6	0.56	0.64	0.38	0.31	0.44	-0.76	-0.91	-0.62

*Scores are standardized. *LL* and *UL* indicate the lower and upper limits of the 95% confidence interval for the mean, respectively. The confidence interval is a plausible range of population means that could have created a sample mean (Cumming, 2014)*.

*The sample size for non-stratregic, prosocial, bi-strategic, and antisocial is 992,992,501,295, respectively*.

Concerning the other possible profile solutions, the three-profile solution consisted of a *prosocial*, *antisocial*, and *non-strategic* group (Supplementary Table S4), and did not identify the theoretically interesting *bi-strategic* group. The five-profile solution consisted of the same profiles as the four-profile solution, with the *antisocial* group being split into a medium and high level (Supplementary Table S5). The sixth profile solution had the same profiles as the five-profile solution, except the *non-strategic* group was split into two, with the two groups differing only slightly in strategy use (Supplementary Table S6). Based on the fit indices and the theoretical considerations, we chose to focus on the four-profile solution.

Thus, we replicated four of the five profiles identified by Hawley, only failing to identify an average profile in any of the profile solutions. However, the cut-points for the profiles tended to be smaller than those utilized in past research, that is, less than 1 SD. There was one important exception to this. The cut-points for the *antisocial* group tended to be more extreme (>1.5 SD) for aggression and rule breaking. Finally, as can be seen in Table 1, the confidence intervals for variables measured at different time points overlapped, suggesting that the composition of the resource control types did not significantly change across time (Research question 2).

We next examined gender differences between profiles. Females were more likely to be characterized as using *prosocial* (*n*_f_ = 699, *n*_m_ = 293) or bi-strategic (*n*_f_ = 337, *n*_m_ = 164) strategies, whereas males were more likely to be characterized as using *non-strategic* (*n*_f_ = 277, *n*_m_ = 715) or *antisocial* (*n*_f_ = 90, *n*_m_ = 205) strategies, *X*^2^[3] = 463.92.

### Profile and Friendship Nominations

We utilized multi-level, negative binomial analyses to predict close friendship nominations using the gender of the friendship nominator, gender of the friendship nominee, profile and developmental period (see analyses section). Hypothesis 1a and 3 propose that profile membership will have the biggest effects amongst opposite sex relationships. This is tested by the three-way interaction involving gender of nominator, gender of the nominee, and profile. Our research question 3 focuses on the extent any effects hold across junior and senior high school and is tested by the four-way interaction involving gender of nominator and nominee, and profile and stage in high school. The *non-strategic* group profile was the contrast group in all analyses, unless otherwise noted.

Hypothesis 1a predicted that *non-strategic* youth would receive the fewest nominations of any group. Main effect tests provided support for this hypothesis: *non-strategic* youth generally received fewer friendship nominations that *prosocial* youth (*B* = 0.51, SE = 0.06, 95% CI [0.39, 0.64]), *bi-strategic* youth (*B* = 0.67, SE = 0.08, [0.52, 0.83]), and *antisocial* youth (*B* = 0.23, SE = 0.08, [0.08, 0.38]). However, consistent with Hypothesis 1b, this effect was qualified by an interaction involving gender × sex of nominator × sex of nominee. The interaction was significant for the *prosocial* group (*B* = 0.43, SE = 0.09, 95% CI [0.24, 0.61]), the *bi-strategic* group (*B* = 0.93, SE = 0.12, [0.69, 1.2]), and the *antisocial* group (*B* = 0.62, SE = 0.16, [0.30, 0.93]).

Figure 1 illustrates these effects (Please see Supplementary Table S8 for results broken down by year). The *non-strategic* group had the fewest opposite sex friends (males nominating females; females nominating males), but did not appear to be as disadvantaged in same sex relationships. The effects appeared to be qualified by developmental period (tested below). Focusing on nominations in junior high, *non-strategics* received fewer opposite sex friendship nominations than any other group (that is, confidence intervals did not overlap). In same sex relationships, the *non-strategics* received fewer nominations than the *prosocial* group and *bi-strategic* groups amongst females, but do not differ in nominations from the *antisocial* group. In male friendships, *non-strategics* received fewer nominations than only from the *prosocial* profile. Thus, Hypothesis 1a is only partially supported amongst same sex peers in junior high.

**Figure 2:**
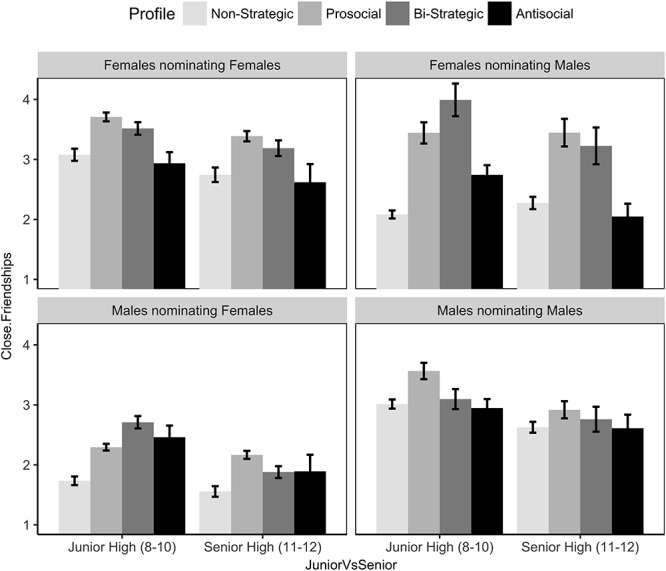
Mean and SE of friendship nominations as a function of profile, gender, and junior versus senior. SE bars represent 95% confidence intervals, a plausible range of population means that could have created the sample means (Cumming, 2014).

We next evaluated the extent that these interaction effects were modified by developmental period. There was no significant interaction involving the *prosocial* group (*B* = -0.03, SE = 0.15, 95% CI [-0.32, 0.26]) or the *antisocial* group (*B* = -0.56, SE = 0.29, [-1.13, 0.001]). However, there was a reliable interaction involving the *bi-strategic* group (*B* = -0.60, SE = 0.18, [-0.96, -0.25]). This effect can be understood by focusing on the opposite sex graphs in Figure 2. Amongst opposite sex relationships, the difference between the *non-strategic* and *bi-strategic* was greater in junior high than senior high. Another way to state this is that *bi-strategic* youth appear to attract fewer opposite sex, close friendship nominations as they get older. This decrease was not as strong in same sex relationships.

Hypothesis 3 posited that *bi-strategics* would be preferred over *prosocials* amongst opposite sex friendships. To test this hypothesis, we re-ran the multilevel analyses with *prosocial*, instead of *non-strategic*, as the contrast group. There was a significant interaction involving *bi-strategic* profile and gender of the nominator and gender of the nominee (*B* = -0.49, SE = 0.12, 95% CI [0.26, -71]), consistent with hypothesis 2b. This interaction was qualified by developmental period (*B* = -0.56, SE = 0.17, [-0.89, -0.23]). Focusing on the opposite sex relationships in Figure 2, *bi-strategics* were favored over prosocial youth in junior high, consistent with hypothesis 2. However, this effect did not hold in senior high, and did not hold in same sex relationships in any developmental period. Figure 1 also illustrates that *bi-strategics* females were favored over *antisocial* females in same sex relationships, providing partial support for hypothesis 2a. However, this effect did not hold in same sex, male relationships.

### Profile, Mental Health, and Self-Esteem

**Figure 3:**
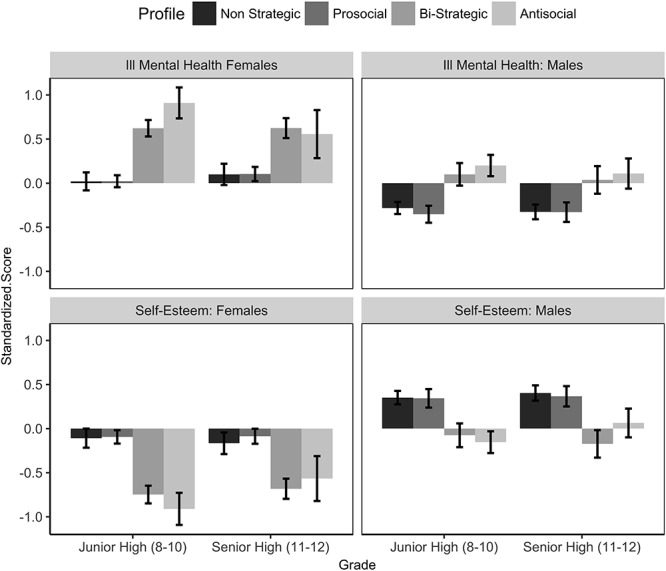
Mental Health and Self-Esteem broken down by Profile and Gender. Error bars represent 95% confidence intervals.

Multi-level linear models predicted self-esteem and mental health utilizing profile, gender and developmental period as predictors. Consistent with Hypothesis 4, youth characterized as *antisocial* were lower than *non-strategics* in self-esteem (*B* = -0.50, SE = 0.070, 95% CI [-0.64, -0.37]) and higher in ill-mental health (*B* = 0.48, SE = 0.07, [0.35, 0.62]). *Bi-strategics* were also found to be lower on both self-esteem (*B* = -0.43, SE = 0.08, [-0.58, -0.28]) and ill-mental health (*B* = 0.37, SE = 0.07, [0.23, 0.52]). However, these effects were qualified by two interactions, as illustrated in Figure 3. First, the effect of profile depended on gender. Being characterized by the *antisocial* profile was associated with worse self-esteem (*B*_anti_ = -0.32, SE = 0.13, 95% CI [-0.57, -0.07]) and mental health (*B*_anti_ = 0.42, SE = 0.13, [0.16, 0.67]) for females compared to males. Further, being *bi-strategic* was associated with somewhat lower self-esteem (*B*_bi_ = -0.21, SE = 0.10, 95% CI [-0.42, -0.007]) for females compared to males. There was a further developmental effect for mental health and antisocial behavior. The *antisocial* profile was associated with worse mental health for females than males in junior high, but this effect was not as strong in senior high (*B* = -0.39, SE = 0.16, [-0.71, -0.07]).

## Discussion

We examined the longitudinal correlates of resource control strategies for friendship nominations and well-being. We utilized a novel approach to clustering joint trajectories (Genolini et al., 2013) and identified four profiles that had been identified previously using human derived cut-offs (Hawley and Little, 2002). These were *bi-strategic*, *non-strategic*, *antisocial*, and *non-strategic*. The statistically derived cut-offs were generally smaller (less than 1 SD) than the human-derived cut-offs (1 SD). There was one important exception. The *antisocial* profile was characterized as being very high in aggression (>1.46 SD) and rule breaking (>1.64 SD). The composition of the profiles was stable across high school.

The consequences of these profiles depended on type of friendship and developmental period. In junior high school (Grades 8–10), *bi-strategic* youth had the highest number of opposite sex, close friendship nominations of any group. This effect replicated for both males and females. However, the pattern changed in senior high. The *bi-strategic* were no longer preferred over prosocial youth, and the *antisocial* were no longer preferred over the *non-strategic youth*. In addition, there appeared to be psychological trade-offs to the different resource control types. *Non-strategic* youth had the fewest opposite sex friendships but had high self-esteem and good mental health relative to the *antisocial* and *bi-strategic* youth. *Prosocial* youth faired the best of all profiles: Throughout high school, they had the highest numbers of same-sex friends and the highest well-being. They did have fewer opposite sex friendships than bi-strategics in junior high, but this disadvantage disappeared by senior high.

### Resource Control in Friendship

Resource control theory suggests that both prosocial and antisocial strategies are utilized in the service of winning resources, which in the present study was conceptualized as friendships. However, not all strategies worked equally well in different social contexts and time periods. In same sex relationships, the *prosocial* group received the most friendship nominations of any profile. The *bi-strategic* never received more nominations than the *prosocial* and the *antisocial* never received more nominations than *non-strategic*, regardless of gender or time period. However, this pattern was quite different in opposite sex friendships, at least in junior high. *Bi-strategic* youth received the most opposite sex friendship nominations of any profile. In addition, youth characterized as *antisocial* were preferred to the *non-strategic* group, despite being nearly 2 SD higher in aggression and rule breaking.

The gender effects in junior high largely line up with our hypotheses, and are consistent with two previous findings. First, socially dominant behaviors, such as the aggression displayed by *bi-strategics*, tend to be less appealing in the same sex compared to the opposite sex (Kerestes and Milanovic, 2007; Ciarrochi and Heaven, 2009). We speculate that this is because social dominance is status threatening within same sex relationships, but not opposite sex relationships. That is, males and females tend to compete more with the same sex than the opposite sex.

Our same-sex friendship findings largely replicate past research, which has shown that antisocial youth tend to be less liked than other youth (van den Broek et al., 2016). Much of the past research has focused on same-sex friendships. However, Pouwels et al. (2018) showed that antisocial behavior (i.e., bullying) is also less liked in the opposite sex. We showed the exact opposite effect: bi-strategic and antisocial youth where preferred by the opposite sex, at least in junior high. There was one key difference between the two studies. The Pouwels et al. bully measure was based on peer ratings, whereas our antisocial measure was based on self-report. Young people may self-report being antisocial but often be perceived by their peers as leaders (Waasdorp et al., 2013), rather than bullies. Future research is needed to examine if friendship nominations are linked to self-versus peer reported antisociality.

The clearest developmental effect involved the youth characterized as *bi-strategic*. In junior high, they received more close friendship nominations from the opposite sex than any other profile. However, from junior high to senior high, *bi-strategic* lost approximately one full opposite sex, friendship nomination. The effect was reliably observed in both males and females. We can only speculate about this intriguing finding. It is possible that *bi-strategic* youth are skilful at hiding some of their more “unsavory” antisocial characteristics. However, as they journey through high school and spend increasing time with their peers, those peers start to detect their negative characteristics. There is clear evidence that greater experience is associated with greater personality detection accuracy (Funder, 1995). Thus, we speculate that youth characterized as *bi-strategic* engage in aversive behaviors that are eventually detected by the opposite sex friends and repel some of them.

### Do Males Pay Attention to Female Empathy?

The findings also appear to address a question raised by past research. Ciarrochi and Heaven (2009) found that females’ ratings of a male’s adjustment were influenced by that male’s level of prosocial traits (e.g., agreeableness), but males’ ratings of female’s adjustment was uninfluenced by their level of prosocial traits. Similarly, Sahdra et al. (2015) found that males nominated empathic males as prosocial but not empathic females, whereas females nominated other empathic males and females as prosocial. This raised an important question: are males unable to detect prosocial traits in females, or do they detect the traits but not utilize them in their judgments? This question was raised again in a later study, when Ciarrochi et al. (2016a) found that females tended to nominate empathic males as friends, but males did not nominate empathic females. Are males insensitive to opposite sex empathy? Or perhaps indifferent?

The present data can begin to answer this question. The main limitation of the past research was that it did not include measures that allowed the assessment of resource control types. The present study provides evidence that males both detect opposite sex empathy and value it, but only sometimes. Males prefer *prosocial* females over *non-strategic* females in both junior and senior high. If they were unable to detect empathy-related behavior or did not care about it, they would make no distinction between *prosocial* and *non-strategic*.

The reason we did not previously observe a link between empathy and male judgments of females was because the effect was masked by males’ preference for *antisocial* females, a subgroup that is low in empathy. In both junior and senior high, males had as many close *antisocial* as *prosocial* female friends. In contrast, females had a clear preference for *prosocial* males in both of these time periods.

### The Resource Control Type Trade-Off

Non-strategic youth do worse than coercive youth (bi-strategic and antisocial) in attracting opposite sex friendships, but do experience higher self-esteem and mental health than coercive youth. This is consistent with what has been found in past adult research (Hawley et al., 2009). Thus, youth can be socially successful but psychologically unhappy.

Antisocial behavior such as aggression and gossip may help youth achieve social dominance and be noticed by the opposite sex but may interfere with their ability to form genuine connections. Perhaps bi-strategic and antisocial youth possess friends the way materialistic people possess objects. Research suggests that when people are successful at their materialistic goals, they are not happier, and can even become less happy (Kasser et al., 2004; Sheldon et al., 2004). Similarly, *bi-strategics* may be more successful than others at “acquiring” friends and popularity, but such success may not bring them genuine connection and need satisfaction.

### Limitations and Future Directions

Future research is needed to examine the mechanisms through which resource control strategies win friends and influence well-being. For example, how does resource control satisfy basic psychological needs, such as need for autonomy, connectedness, and competence (Ryan and Deci, 2017)? Youth who use coercive control strategies (*bi-strategic and antisocial*) may get their competence needs met through higher levels of popularity, but fail to get their connection needs met.

Theories of aggression and empathy suggest interesting future research directions. For example, the GAM (Allen et al., 2018) suggests that internal states like frustration can lead to either thoughtful or impulsive action. Are the bi-strategic youth more likely to use thoughtful forms of aggression, given they are likely to be aware of the influence of impulsive aggression on others? Is strategic aggression more effective at attracting opposite sex friends that reactive aggression? Future research should examine these possibilities.

Prosocial youth tended to attract more same sex friendships than any other group. Future research is needed to examine the motivation behind their empathic behavior. Zaki (2014) proposed that empathic responses are more likely to occur when a young person is motivated to build affiliation, whereas empathic avoidance is more likely to occur when the youth is motivated to avoid pain or empathic distress. We would speculate that *prosocial* youth avoid empathic distress by engaging in low levels of antisocial behavior, which may bring harm to the other and elicit distress in themselves. In contrast, *bi-strategic* youth may be less motivated to avoid the empathic distress associated with aggressive behavior. This could explain why bi-strategic youth, in our study, report being both more willing to engage in aggressive behavior and more likely to experience poor mental health. Future research is needed to investigate these interesting possibilities.

The present study represents only a start to a potentially exciting line of inquiry. Future research is needed to examine not just the form of the prosocial and antisocial behavior, as was done in the present study, but also the function and effectiveness of that behavior. If a young person acts aggressively toward a friend out of fear, does this have different consequences than when she acts aggressively toward a friend in order to get her needs met (Little et al., 2003)? What is the consequence for friendship for someone who uses an ineffective form of empathy, e.g., one that increases empathic distress without having positive social consequences?

## Ethics Statement

This study was carried out in accordance with the recommendations of ACU Ethics Committee with written informed consent from all subjects. All subjects gave written informed consent in accordance with the Declaration of Helsinki. The protocol was approved by the ACU Ethics Committee.

## Author Contributions

JC and BS contributed to the data analyses and writing of the manuscript. PH and ED contributed to the writing of the manuscript.

## Conflict of Interest Statement

The authors declare that the research was conducted in the absence of any commercial or financial relationships that could be construed as a potential conflict of interest.
