# Supplementary material for: The Upsides and Downsides of the Dark Side: A Longitudinal Study Into the Role of Prosocial and Antisocial Strategies in Close Friendship Formation
Source: Front Psychol. 2019 Feb 19;10:114. doi: 10.3389/fpsyg.2019.00114 (PMC6401596; doi:10.3389/fpsyg.2019.00114)
Supplement: Supplementary file 1 [file Table_1.docx]

# Table S1. Means, standard deviations, and standard errors for study variables

|  |  | **Males** |  |  | **Females** |  | **Female vs. Male** |
| --- | --- | --- | --- | --- | --- | --- | --- |
|  | **Mean** | **SD** | **SE** | **Means** | **SD** | **SE** | **Cohen's d** |
| Sex of friendship nominator |  |  |  |  |  |  |  |
| Male Grade 8 | 2.96 | 2.31 | 0.07 | 2.32 | 2.84 | 0.11 | 0.25 |
| Female Grade 8 | 2.59 | 3.4 | 0.14 | 3.46 | 2.29 | 0.07 | -0.3 |
| Male Grade 9 | 3.24 | 2.54 | 0.08 | 2.67 | 3 | 0.12 | 0.21 |
| Female Grade 9 | 3 | 3.57 | 0.14 | 3.68 | 2.17 | 0.07 | -0.23 |
| Male Grade 10 | 3.3 | 2.31 | 0.07 | 2.63 | 2.98 | 0.12 | 0.25 |
| Female Grade 10 | 2.8 | 3.28 | 0.13 | 3.68 | 2.18 | 0.07 | -0.32 |
| Male Grade 11 | 3.02 | 2.23 | 0.08 | 2.33 | 2.66 | 0.11 | 0.28 |
| Female Grade 11 | 3.04 | 3.21 | 0.14 | 3.62 | 2.15 | 0.07 | -0.21 |
| Male Grade 12 | 3.24 | 2.12 | 0.08 | 2.29 | 2.54 | 0.11 | 0.41 |
| Female Grade 12 | 3 | 3.13 | 0.15 | 3.38 | 2 | 0.07 | -0.14 |
| Aggression |  |  |  |  |  |  |  |
| Grade 8 | 0.43 | 0.36 | 0.01 | 0.41 | 0.34 | 0.01 | 0.06 |
| Grade 9 | 0.46 | 0.41 | 0.01 | 0.44 | 0.38 | 0.01 | 0.05 |
| Grade 10 | 0.41 | 0.35 | 0.01 | 0.4 | 0.33 | 0.01 | 0.03 |
| Grade 11 | 0.4 | 0.35 | 0.01 | 0.38 | 0.32 | 0.01 | 0.06 |
| Rule Breaking |  |  |  |  |  |  |  |
| Grade 8 | 0.37 | 0.3 | 0.01 | 0.29 | 0.28 | 0.01 | 0.28 |
| Grade 9 | 0.46 | 0.39 | 0.01 | 0.37 | 0.36 | 0.01 | 0.24 |
| Grade 10 | 0.44 | 0.34 | 0.01 | 0.36 | 0.32 | 0.01 | 0.24 |
| Grade 11 | 0.46 | 0.35 | 0.01 | 0.36 | 0.31 | 0.01 | 0.3 |
| Affective Empathy |  |  |  |  |  |  |  |
| Grade 8 | 2.83 | 0.51 | 0.02 | 3.4 | 0.51 | 0.02 | -1.12 |
| Grade 9 | 3.01 | 0.62 | 0.02 | 3.67 | 0.62 | 0.02 | -1.06 |
| Grade 10 | 3.09 | 0.65 | 0.02 | 3.71 | 0.62 | 0.02 | -0.98 |
| Grade 11 | 3.17 | 0.64 | 0.02 | 3.73 | 0.63 | 0.02 | -0.88 |
| Cognitive Empathy |  |  |  |  |  |  |  |
| Grade 8 | 3.77 | 0.56 | 0.02 | 4.06 | 0.54 | 0.02 | -0.53 |
| Grade 9 | 3.83 | 0.63 | 0.02 | 4.18 | 0.55 | 0.02 | -0.59 |
| Grade 10 | 3.92 | 0.6 | 0.02 | 4.19 | 0.52 | 0.02 | -0.48 |
| Grade 11 | 3.94 | 0.59 | 0.02 | 4.19 | 0.55 | 0.02 | -0.44 |
| Self-Esteem |  |  |  |  |  |  |  |
| Grade 8 | 0.76 | 0.23 | 0.01 | 0.65 | 0.28 | 0.01 | 0.43 |
| Grade 9 | 0.74 | 0.24 | 0.01 | 0.59 | 0.29 | 0.01 | 0.56 |
| Grade 10 | 0.74 | 0.25 | 0.01 | 0.57 | 0.3 | 0.01 | 0.62 |
| Grade 11 | 0.72 | 0.26 | 0.01 | 0.56 | 0.29 | 0.01 | 0.58 |
| Grade 12 | 0.73 | 0.24 | 0.01 | 0.59 | 0.28 | 0.01 | 0.54 |
| Mental Health |  |  |  |  |  |  |  |
| Grade 8 | 1.76 | 0.49 | 0.02 | 1.95 | 0.54 | 0.02 | -0.37 |
| Grade 9 | 1.85 | 0.53 | 0.02 | 2.08 | 0.59 | 0.02 | -0.41 |
| Grade 10 | 1.85 | 0.5 | 0.02 | 2.11 | 0.58 | 0.02 | -0.48 |
| Grade 11 | 1.93 | 0.52 | 0.02 | 2.19 | 0.61 | 0.02 | -0.46 |
| Grade 12 | 1.97 | 0.5 | 0.02 | 2.25 | 0.6 | 0.02 | -0.51 |
