# Supplementary material for: The Upsides and Downsides of the Dark Side: A Longitudinal Study Into the Role of Prosocial and Antisocial Strategies in Close Friendship Formation
Source: Front Psychol. 2019 Feb 19;10:114. doi: 10.3389/fpsyg.2019.00114 (PMC6401596; doi:10.3389/fpsyg.2019.00114)
Supplement: Supplementary file 3 [file Table_3.docx]

# Table S3: Relationship (spearman) between cognitive and affective, and close friendship nominations from Grade 8 to Grade 12

|  | AEmp8 | AEmp9 | AEmp10 | AEmp11 | CEmp8 | CEmp9 | CEmp10 | CEmp11 |
| --- | --- | --- | --- | --- | --- | --- | --- | --- |
| **Nominator** | |  |  | **Male receiving nominations** | | |  |  |
| Male 8 | 0.06 | 0.04 | 0.08 | 0.03 | 0.11 | 0.07 | 0.13 | 0.03 |
| Female 8 | 0.12 | 0.16 | 0.16 | 0.08 | 0.16 | 0.12 | 0.19 | 0.15 |
| Male 9 | 0.03 | 0.06 | 0.05 | 0.02 | 0.11 | 0.09 | 0.11 | 0.01 |
| Female 9 | 0.15 | 0.18 | 0.21 | 0.11 | 0.19 | 0.2 | 0.2 | 0.14 |
| In | 0.06 | 0.11 | 0.1 | 0.06 | 0.1 | 0.06 | 0.13 | 0.03 |
| Female 10 | 0.13 | 0.21 | 0.19 | 0.21 | 0.19 | 0.24 | 0.27 | 0.21 |
| Male 11 | 0.16 | 0.06 | 0.09 | 0.1 | 0.08 | 0.11 | 0.09 | 0.09 |
| Female 11 | 0.12 | 0.16 | 0.2 | 0.22 | 0.14 | 0.2 | 0.24 | 0.2 |
| Male 12 | 0.01 | -0.01 | 0.1 | 0.04 | 0.08 | 0.02 | 0.06 | 0.07 |
| Female 12 | 0.15 | 0.1 | 0.17 | 0.19 | 0.2 | 0.22 | 0.22 | 0.21 |
|  |  |  |  | **Female receiving nominations** | | | |  |
| Male 8 | 0.08 | 0.06 | -0.05 | -0.01 | 0.16 | 0.06 | 0.09 | 0.09 |
| Female 8 | 0.1 | 0.07 | 0.04 | 0.03 | 0.09 | 0.04 | 0.08 | 0.04 |
| Male 9 | 0.06 | 0.11 | -0.01 | 0.03 | 0.13 | 0.12 | 0.12 | 0.07 |
| Female 9 | 0.06 | 0.07 | 0.04 | 0.03 | 0.03 | 0.07 | 0.01 | 0.08 |
| Male 10 | 0.04 | 0.08 | 0.02 | -0.02 | 0.06 | 0.15 | 0.09 | 0.06 |
| Female 10 | 0.08 | 0.05 | 0.07 | 0.01 | 0.02 | 0 | 0.03 | 0 |
| Male 11 | 0.03 | 0.06 | 0.05 | 0.07 | 0.08 | 0.13 | 0.07 | 0.12 |
| Female 11 | 0.03 | 0.01 | 0.07 | 0.05 | 0.03 | 0.02 | 0.06 | 0.07 |
| Male 12 | 0.03 | 0.05 | 0.02 | -0.02 | 0.07 | 0.13 | 0.09 | 0.01 |
| Female 12 | 0.06 | 0.01 | -0.03 | 0.04 | -0.01 | 0.05 | 0.02 | 0.05 |

**Note:** shaded areas represent opposite sex relationships
