# Supplementary material for: The Upsides and Downsides of the Dark Side: A Longitudinal Study Into the Role of Prosocial and Antisocial Strategies in Close Friendship Formation
Source: Front Psychol. 2019 Feb 19;10:114. doi: 10.3389/fpsyg.2019.00114 (PMC6401596; doi:10.3389/fpsyg.2019.00114)
Supplement: Supplementary file 4 [file Table_4.docx]

# Table S4: Characteristics of the three profile solution, as identified in non-parametric joint trajectory cluster analysis (Grade 8 to 11)

## Grade 8

| Aggression | | | | |
| --- | --- | --- | --- | --- |
| *Profile* | *M* | *LL* | *UL* | *SD* |
| Prosocial | -0.27 | -0.31 | -0.23 | 0.69 |
| Non-Strategic | -0.32 | -0.36 | -0.28 | 0.69 |
| Antisocial | 1.30 | 1.20 | 1.39 | 1.12 |
| Rule Breaking | | | | |
| *Profile* | *M* | *LL* | *UL* | *SD* |
| Prosocial | -0.33 | -0.37 | -0.30 | 0.62 |
| Non-Strategic | -0.25 | -0.29 | -0.22 | 0.61 |
| Antisocial | 1.32 | 1.21 | 1.43 | 1.27 |
| Affective Empathy | | | | |
| *Profile* | *M* | *LL* | *UL* | *SD* |
| Prosocial | 0.60 | 0.56 | 0.65 | 0.80 |
| Non-Strategic | -0.53 | -0.58 | -0.48 | 0.80 |
| Antisocial | -0.25 | -0.35 | -0.16 | 1.05 |
| Cognitive Empathy | | | | |
| *Profile* | *M* | *LL* | *UL* | *SD* |
| Prosocial | 0.56 | 0.52 | 0.60 | 0.76 |
| Non-Strategic | -0.50 | -0.55 | -0.44 | 0.85 |
| Antisocial | -0.24 | -0.34 | -0.14 | 1.13 |

Note: *N* for Prosocial, non-strategic, and antisocial is 1186,1084, and 510, respectively

## Grade 9

Means and standard deviations for outcome as a function of a 3(Profile) X 4(Type) design

| Aggression | | | | |
| --- | --- | --- | --- | --- |
| *Profile* | *M* | *LL* | *UL* | *SD* |
| Prosocial | -0.28 | -0.32 | -0.25 | 0.66 |
| Non-Strategic | -0.38 | -0.42 | -0.35 | 0.60 |
| Antisocial | 1.47 | 1.38 | 1.56 | 1.02 |
| Rule Breaking | | | | |
| *Profile* | *M* | *LL* | *UL* | *SD* |
| Prosocial | -0.35 | -0.38 | -0.31 | 0.62 |
| Non-Strategic | -0.32 | -0.35 | -0.28 | 0.54 |
| Antisocial | 1.48 | 1.38 | 1.58 | 1.12 |
| Affective Empathy | | | | |
| *Profile* | *M* | *LL* | *UL* | *SD* |
| Prosocial | 0.70 | 0.66 | 0.74 | 0.70 |
| Non-Strategic | -0.60 | -0.65 | -0.56 | 0.76 |
| Antisocial | -0.33 | -0.42 | -0.24 | 1.04 |
| Cognitive Empathy | | | | |
| *Profile* | *M* | *LL* | *UL* | *SD* |
| Prosocial | 0.62 | 0.58 | 0.65 | 0.67 |
| Non-Strategic | -0.50 | -0.55 | -0.45 | 0.84 |
| Antisocial | -0.35 | -0.45 | -0.25 | 1.18 |

## Grade 10

Means and standard deviations for outcome as a function of a 3(Profile) X 4(Type) design

| Aggression | | | | |
| --- | --- | --- | --- | --- |
| *Profile* | *M* | *LL* | *UL* | *SD* |
| Prosocial | -0.27 | -0.30 | -0.23 | 0.67 |
| Non-Strategic | -0.39 | -0.42 | -0.35 | 0.59 |
| Antisocial | 1.44 | 1.35 | 1.53 | 1.07 |
| Rule Breaking | | | | |
| *Profile* | *M* | *LL* | *UL* | *SD* |
| Prosocial | -0.35 | -0.38 | -0.31 | 0.63 |
| Non-Strategic | -0.32 | -0.35 | -0.28 | 0.55 |
| Antisocial | 1.48 | 1.39 | 1.58 | 1.10 |
| Affective Empathy | | | | |
| *Profile* | *M* | *LL* | *UL* | *SD* |
| Prosocial | 0.70 | 0.67 | 0.74 | 0.70 |
| Non-Strategic | -0.60 | -0.64 | -0.55 | 0.75 |
| Antisocial | -0.36 | -0.45 | -0.27 | 1.05 |
| Cognitive Empathy | | | | |
| *Profile* | *M* | *LL* | *UL* | *SD* |
| Prosocial | 0.61 | 0.57 | 0.65 | 0.66 |
| Non-Strategic | -0.51 | -0.56 | -0.46 | 0.84 |
| Antisocial | -0.33 | -0.44 | -0.23 | 1.20 |

## Grade 11

Means and standard deviations for outcome as a function of a 3(Profile) X 4(Type) design

| Aggression | | | | |
| --- | --- | --- | --- | --- |
| *Profile* | *M* | *LL* | *UL* | *SD* |
| Prosocial | -0.27 | -0.31 | -0.23 | 0.68 |
| Non-Strategic | -0.35 | -0.39 | -0.31 | 0.67 |
| Antisocial | 1.37 | 1.28 | 1.46 | 1.06 |
| Rule Breaking | | | | |
| *Profile* | *M* | *LL* | *UL* | *SD* |
| Prosocial | -0.36 | -0.40 | -0.33 | 0.63 |
| Non-Strategic | -0.27 | -0.31 | -0.23 | 0.66 |
| Antisocial | 1.42 | 1.33 | 1.51 | 1.06 |
| Affective Empathy | | | | |
| *Profile* | *M* | *LL* | *UL* | *SD* |
| Prosocial | 0.69 | 0.65 | 0.73 | 0.72 |
| Non-Strategic | -0.58 | -0.63 | -0.54 | 0.77 |
| Antisocial | -0.35 | -0.44 | -0.26 | 1.03 |
| Cognitive Empathy | | | | |
| *Profile* | *M* | *LL* | *UL* | *SD* |
| Prosocial | 0.61 | 0.57 | 0.65 | 0.67 |
| Non-Strategic | -0.49 | -0.54 | -0.44 | 0.85 |
| Antisocial | -0.36 | -0.46 | -0.26 | 1.17 |
