# Supplementary material for: The Upsides and Downsides of the Dark Side: A Longitudinal Study Into the Role of Prosocial and Antisocial Strategies in Close Friendship Formation
Source: Front Psychol. 2019 Feb 19;10:114. doi: 10.3389/fpsyg.2019.00114 (PMC6401596; doi:10.3389/fpsyg.2019.00114)
Supplement: Supplementary file 5 [file Table_5.docx]

# Table S5: Characteristics of the five-profile solution, as identified in non-parametric joint trajectory cluster analysis (Grade 8 to 11)

## Grade 8

| Aggression | | | | |
| --- | --- | --- | --- | --- |
| *Profile* | *M* | *LL* | *UL* | *SD* |
| Non-Strategic | -0.51 | -0.55 | -0.47 | 0.55 |
| Prosocial | -0.52 | -0.55 | -0.49 | 0.48 |
| Medium Antisocial | 0.51 | 0.43 | 0.59 | 0.87 |
| Bi-Strategic | 0.65 | 0.58 | 0.73 | 0.83 |
| Antisocial | 1.90 | 1.70 | 2.10 | 1.29 |
| Rule Breaking | | | | |
| *Profile* | *M* | *LL* | *UL* | *SD* |
| Non-Strategic | -0.42 | -0.45 | -0.38 | 0.53 |
| Prosocial | -0.53 | -0.57 | -0.50 | 0.45 |
| Medium Antisocial | 0.49 | 0.42 | 0.57 | 0.85 |
| Bi-Strategic | 0.41 | 0.34 | 0.48 | 0.78 |
| Antisocial | 2.21 | 1.98 | 2.43 | 1.47 |
| Affective Empathy | | | | |
| *Profile* | *M* | *LL* | *UL* | *SD* |
| Non-Strategic | -0.50 | -0.55 | -0.45 | 0.77 |
| Prosocial | 0.61 | 0.56 | 0.67 | 0.77 |
| Medium Antisocial | -0.63 | -0.71 | -0.56 | 0.87 |
| Bi-Strategic | 0.65 | 0.58 | 0.72 | 0.77 |
| Antisocial | -0.32 | -0.49 | -0.14 | 1.15 |
| Cognitive Empathy | | | | |
| *Profile* | *M* | *LL* | *UL* | *SD* |
| Non-Strategic | -0.30 | -0.36 | -0.25 | 0.84 |
| Prosocial | 0.58 | 0.52 | 0.63 | 0.75 |
| Medium Antisocial | -0.74 | -0.83 | -0.66 | 0.94 |
| Bi-Strategic | 0.44 | 0.37 | 0.51 | 0.79 |
| Antisocial | -0.28 | -0.48 | -0.07 | 1.32 |

Note: *N* for Non-strategic, Prosocial, Medium Antisocial, Bi-Strategic, and Antisocial is 867,807,476,464,166, respectively
