# Supplementary material for: The Upsides and Downsides of the Dark Side: A Longitudinal Study Into the Role of Prosocial and Antisocial Strategies in Close Friendship Formation
Source: Front Psychol. 2019 Feb 19;10:114. doi: 10.3389/fpsyg.2019.00114 (PMC6401596; doi:10.3389/fpsyg.2019.00114)
Supplement: Supplementary file 6 [file Table_6.docx]

# Table S6: Characteristics of the six-profile solution, as identified in non-parametric joint trajectory cluster analysis (Grade 8 to 11)

## Grade 8

| Aggression | | | | |
| --- | --- | --- | --- | --- |
| *Profile* | *M* | *LL* | *UL* | *SD* |
| Prosocial | -0.47 | -0.51 | -0.43 | 0.50 |
| Non-strategic | -0.32 | -0.38 | -0.27 | 0.66 |
| Non-strategic2 | -0.54 | -0.59 | -0.50 | 0.55 |
| Bi-Strategic | 0.75 | 0.67 | 0.84 | 0.88 |
| Medium Antisocial | 0.77 | 0.68 | 0.87 | 0.94 |
| High antisocial | 1.96 | 1.71 | 2.21 | 1.38 |
| Rule Breaking | | | | |
| *Profile* | *M* | *LL* | *UL* | *SD* |
| Prosocial | -0.50 | -0.54 | -0.47 | 0.47 |
| Non-strategic | -0.25 | -0.30 | -0.21 | 0.58 |
| Non-strategic2 | -0.47 | -0.51 | -0.43 | 0.52 |
| Bi-Strategic | 0.52 | 0.43 | 0.60 | 0.86 |
| Medium Antisocial | 0.79 | 0.69 | 0.89 | 1.02 |
| High antisocial | 2.25 | 1.97 | 2.54 | 1.57 |
| Affective Empathy | | | | |
| *Profile* | *M* | *LL* | *UL* | *SD* |
| Prosocial | 0.77 | 0.71 | 0.82 | 0.71 |
| Non-strategic | -0.46 | -0.53 | -0.40 | 0.81 |
| Non-strategic2 | -0.45 | -0.51 | -0.38 | 0.76 |
| Bi-Strategic | 0.67 | 0.59 | 0.74 | 0.77 |
| Medium Antisocial | -0.64 | -0.73 | -0.55 | 0.86 |
| High antisocial | -0.41 | -0.63 | -0.19 | 1.19 |
| Cognitive Empathy | | | | |
| *Profile* | *M* | *LL* | *UL* | *SD* |
| Prosocial | 0.52 | 0.46 | 0.58 | 0.77 |
| Non-strategic | -0.88 | -0.94 | -0.82 | 0.75 |
| Non-strategic2 | 0.33 | 0.28 | 0.39 | 0.70 |
| Bi-Strategic | 0.49 | 0.41 | 0.57 | 0.80 |
| Medium Antisocial | -0.53 | -0.62 | -0.43 | 0.95 |
| High antisocial | -0.44 | -0.68 | -0.19 | 1.37 |

Note: Sample sizes for the profiles are 711, 581, 578, 417, 375, and 118, respectively

## Grade 9

| Aggression | | | | |
| --- | --- | --- | --- | --- |
| *Profile* | *M* | *LL* | *UL* | *SD* |
| Prosocial | -0.51 | -0.54 | -0.48 | 0.44 |
| Non-strategic | -0.41 | -0.45 | -0.36 | 0.54 |
| Non-strategic2 | -0.59 | -0.63 | -0.55 | 0.48 |
| Bi-Strategic | 0.83 | 0.76 | 0.91 | 0.78 |
| Medium Antisocial | 0.76 | 0.68 | 0.84 | 0.76 |
| High antisocial | 2.59 | 2.42 | 2.76 | 0.92 |
| Rule Breaking | | | | |
| *Profile* | *M* | *LL* | *UL* | *SD* |
| Prosocial | -0.55 | -0.58 | -0.52 | 0.44 |
| Non-strategic | -0.33 | -0.37 | -0.29 | 0.49 |
| Non-strategic2 | -0.52 | -0.56 | -0.49 | 0.46 |
| Bi-Strategic | 0.65 | 0.57 | 0.73 | 0.81 |
| Medium Antisocial | 0.76 | 0.68 | 0.84 | 0.79 |
| High antisocial | 2.79 | 2.60 | 2.98 | 1.04 |
| Affective Empathy | | | | |
| *Profile* | *M* | *LL* | *UL* | *SD* |
| Prosocial | 0.89 | 0.85 | 0.93 | 0.56 |
| Non-strategic | -0.53 | -0.59 | -0.47 | 0.77 |
| Non-strategic2 | -0.49 | -0.55 | -0.43 | 0.71 |
| Bi-Strategic | 0.74 | 0.68 | 0.81 | 0.68 |
| Medium Antisocial | -0.73 | -0.81 | -0.65 | 0.77 |
| High antisocial | -0.60 | -0.82 | -0.39 | 1.18 |
| Cognitive Empathy | | | | |
| *Profile* | *M* | *LL* | *UL* | *SD* |
| Prosocial | 0.57 | 0.52 | 0.62 | 0.67 |
| Non-strategic | -0.92 | -0.97 | -0.86 | 0.68 |
| Non-strategic2 | 0.40 | 0.35 | 0.45 | 0.66 |
| Bi-Strategic | 0.52 | 0.45 | 0.59 | 0.72 |
| Medium Antisocial | -0.59 | -0.68 | -0.50 | 0.89 |
| High antisocial | -0.78 | -1.08 | -0.48 | 1.64 |

## Grade 10

| Aggression | | | | |
| --- | --- | --- | --- | --- |
| *Profile* | *M* | *LL* | *UL* | *SD* |
| Prosocial | -0.47 | -0.51 | -0.44 | 0.48 |
| Non-strategic | -0.45 | -0.49 | -0.41 | 0.49 |
| Non-strategic2 | -0.56 | -0.60 | -0.52 | 0.49 |
| Bi-Strategic | 0.78 | 0.70 | 0.85 | 0.77 |
| Medium Antisocial | 0.77 | 0.70 | 0.85 | 0.76 |
| High antisocial | 2.64 | 2.46 | 2.82 | 0.99 |
| Rule Breaking | | | | |
| *Profile* | *M* | *LL* | *UL* | *SD* |
| Prosocial | -0.54 | -0.58 | -0.51 | 0.45 |
| Non-strategic | -0.34 | -0.38 | -0.30 | 0.48 |
| Non-strategic2 | -0.52 | -0.56 | -0.49 | 0.46 |
| Bi-Strategic | 0.63 | 0.55 | 0.71 | 0.84 |
| Medium Antisocial | 0.79 | 0.71 | 0.86 | 0.74 |
| High antisocial | 2.81 | 2.63 | 2.99 | 0.99 |
| Affective Empathy | | | | |
| *Profile* | *M* | *LL* | *UL* | *SD* |
| Prosocial | 0.94 | 0.90 | 0.98 | 0.54 |
| Non-strategic | -0.52 | -0.58 | -0.46 | 0.76 |
| Non-strategic2 | -0.51 | -0.56 | -0.45 | 0.69 |
| Bi-Strategic | 0.71 | 0.65 | 0.77 | 0.63 |
| Medium Antisocial | -0.77 | -0.85 | -0.69 | 0.79 |
| High antisocial | -0.60 | -0.82 | -0.39 | 1.17 |
| Cognitive Empathy | | | | |
| *Profile* | *M* | *LL* | *UL* | *SD* |
| Prosocial | 0.58 | 0.53 | 0.63 | 0.67 |
| Non-strategic | -0.91 | -0.96 | -0.85 | 0.70 |
| Non-strategic2 | 0.38 | 0.32 | 0.43 | 0.68 |
| Bi-Strategic | 0.51 | 0.45 | 0.58 | 0.67 |
| Medium Antisocial | -0.64 | -0.73 | -0.54 | 0.95 |
| High antisocial | -0.63 | -0.91 | -0.35 | 1.54 |

## Grade 11

| Aggression | | | | |
| --- | --- | --- | --- | --- |
| *Profile* | *M* | *LL* | *UL* | *SD* |
| Prosocial | -0.48 | -0.52 | -0.45 | 0.48 |
| Non-strategic | -0.45 | -0.49 | -0.41 | 0.53 |
| Non-strategic2 | -0.52 | -0.57 | -0.48 | 0.57 |
| Bi-Strategic | 0.70 | 0.63 | 0.77 | 0.74 |
| Medium Antisocial | 0.85 | 0.77 | 0.93 | 0.78 |
| High antisocial | 2.51 | 2.31 | 2.72 | 1.13 |
| Rule Breaking | | | | |
| *Profile* | *M* | *LL* | *UL* | *SD* |
| Prosocial | -0.55 | -0.59 | -0.52 | 0.48 |
| Non-strategic | -0.34 | -0.38 | -0.29 | 0.54 |
| Non-strategic2 | -0.49 | -0.53 | -0.44 | 0.55 |
| Bi-Strategic | 0.55 | 0.47 | 0.62 | 0.79 |
| Medium Antisocial | 0.90 | 0.82 | 0.98 | 0.78 |
| High antisocial | 2.61 | 2.42 | 2.81 | 1.06 |
| Affective Empathy | | | | |
| *Profile* | *M* | *LL* | *UL* | *SD* |
| Prosocial | 0.90 | 0.86 | 0.94 | 0.58 |
| Non-strategic | -0.47 | -0.53 | -0.41 | 0.76 |
| Non-strategic2 | -0.48 | -0.54 | -0.42 | 0.75 |
| Bi-Strategic | 0.64 | 0.57 | 0.71 | 0.70 |
| Medium Antisocial | -0.78 | -0.86 | -0.69 | 0.83 |
| High antisocial | -0.52 | -0.74 | -0.31 | 1.17 |
| Cognitive Empathy | | | | |
| *Profile* | *M* | *LL* | *UL* | *SD* |
| Prosocial | 0.58 | 0.53 | 0.63 | 0.67 |
| Non-strategic | -0.83 | -0.88 | -0.77 | 0.70 |
| Non-strategic2 | 0.36 | 0.30 | 0.41 | 0.70 |
| Bi-Strategic | 0.51 | 0.44 | 0.58 | 0.71 |
| Medium Antisocial | -0.74 | -0.84 | -0.64 | 0.99 |
| High antisocial | -0.59 | -0.86 | -0.32 | 1.47 |
