# Supplementary material for: The Upsides and Downsides of the Dark Side: A Longitudinal Study Into the Role of Prosocial and Antisocial Strategies in Close Friendship Formation
Source: Front Psychol. 2019 Feb 19;10:114. doi: 10.3389/fpsyg.2019.00114 (PMC6401596; doi:10.3389/fpsyg.2019.00114)
Supplement: Supplementary file 7 [file Table_7.docx]

# Table S7: Four profile solution utilizing k-medians instead of k-means clustering, broken down by year

## Grade 8

| Aggression | | | | |
| --- | --- | --- | --- | --- |
| *Profile* | *M* | *LL* | *UL* | *SD* |
| Non-Strategic | -0.44 | -0.48 | -0.40 | 0.61 |
| Bi-Strategic | 0.34 | 0.28 | 0.40 | 0.75 |
| Prosocial | -0.63 | -0.66 | -0.60 | 0.41 |
| Antisocial | 1.20 | 1.10 | 1.30 | 1.15 |
| Rule Breaking | | | | |
| *Profile* | *M* | *LL* | *UL* | *SD* |
| Non-Strategic | -0.34 | -0.38 | -0.31 | 0.57 |
| Bi-Strategic | 0.12 | 0.07 | 0.18 | 0.71 |
| Prosocial | -0.61 | -0.64 | -0.58 | 0.41 |
| Antisocial | 1.25 | 1.14 | 1.36 | 1.29 |
| Affective Empathy | | | | |
| *Profile* | *M* | *LL* | *UL* | *SD* |
| Non-Strategic | -0.53 | -0.58 | -0.48 | 0.78 |
| Bi-Strategic | 0.65 | 0.59 | 0.71 | 0.74 |
| Prosocial | 0.59 | 0.52 | 0.65 | 0.83 |
| Antisocial | -0.45 | -0.54 | -0.37 | 1.00 |
| Cognitive Empathy | | | | |
| *Profile* | *M* | *LL* | *UL* | *SD* |
| Non-Strategic | -0.45 | -0.50 | -0.40 | 0.87 |
| Bi-Strategic | 0.29 | 0.23 | 0.35 | 0.79 |
| Prosocial | 0.73 | 0.68 | 0.79 | 0.73 |
| Antisocial | -0.35 | -0.45 | -0.25 | 1.12 |

## Grade 9

| Aggression | | | | |
| --- | --- | --- | --- | --- |
| *Profile* | *M* | *LL* | *UL* | *SD* |
| Non-Strategic | -0.49 | -0.52 | -0.45 | 0.53 |
| Bi-Strategic | 0.33 | 0.28 | 0.39 | 0.70 |
| Prosocial | -0.67 | -0.70 | -0.64 | 0.36 |
| Antisocial | 1.35 | 1.25 | 1.44 | 1.06 |
| Rule Breaking | | | | |
| *Profile* | *M* | *LL* | *UL* | *SD* |
| Non-Strategic | -0.40 | -0.43 | -0.36 | 0.50 |
| Bi-Strategic | 0.15 | 0.09 | 0.20 | 0.69 |
| Prosocial | -0.66 | -0.70 | -0.63 | 0.39 |
| Antisocial | 1.39 | 1.29 | 1.49 | 1.15 |
| Affective Empathy | | | | |
| *Profile* | *M* | *LL* | *UL* | *SD* |
| Non-Strategic | -0.60 | -0.65 | -0.55 | 0.74 |
| Bi-Strategic | 0.72 | 0.66 | 0.77 | 0.68 |
| Prosocial | 0.71 | 0.65 | 0.76 | 0.71 |
| Antisocial | -0.54 | -0.62 | -0.45 | 0.96 |
| Cognitive Empathy | | | | |
| *Profile* | *M* | *LL* | *UL* | *SD* |
| Non-Strategic | -0.47 | -0.53 | -0.42 | 0.84 |
| Bi-Strategic | 0.38 | 0.32 | 0.43 | 0.73 |
| Prosocial | 0.79 | 0.75 | 0.84 | 0.60 |
| Antisocial | -0.47 | -0.57 | -0.37 | 1.16 |

## Grade 10

| Aggression | | | | |
| --- | --- | --- | --- | --- |
| *Profile* | *M* | *LL* | *UL* | *SD* |
| Non-Strategic | -0.50 | -0.53 | -0.47 | 0.51 |
| Bi-Strategic | 0.33 | 0.28 | 0.39 | 0.67 |
| Prosocial | -0.65 | -0.68 | -0.62 | 0.40 |
| Antisocial | 1.34 | 1.24 | 1.43 | 1.10 |
| Rule Breaking | | | | |
| *Profile* | *M* | *LL* | *UL* | *SD* |
| Non-Strategic | -0.41 | -0.44 | -0.37 | 0.50 |
| Bi-Strategic | 0.16 | 0.11 | 0.22 | 0.70 |
| Prosocial | -0.67 | -0.70 | -0.64 | 0.38 |
| Antisocial | 1.39 | 1.30 | 1.49 | 1.13 |
| Affective Empathy | | | | |
| *Profile* | *M* | *LL* | *UL* | *SD* |
| Non-Strategic | -0.58 | -0.63 | -0.54 | 0.75 |
| Bi-Strategic | 0.69 | 0.64 | 0.74 | 0.65 |
| Prosocial | 0.73 | 0.67 | 0.78 | 0.73 |
| Antisocial | -0.56 | -0.65 | -0.48 | 0.97 |
| Cognitive Empathy | | | | |
| *Profile* | *M* | *LL* | *UL* | *SD* |
| Non-Strategic | -0.47 | -0.52 | -0.42 | 0.85 |
| Bi-Strategic | 0.35 | 0.29 | 0.40 | 0.70 |
| Prosocial | 0.80 | 0.76 | 0.85 | 0.61 |
| Antisocial | -0.47 | -0.57 | -0.37 | 1.17 |

## Grade 11

| Aggression | | | | |
| --- | --- | --- | --- | --- |
| *Profile* | *M* | *LL* | *UL* | *SD* |
| Non-Strategic | -0.48 | -0.51 | -0.44 | 0.57 |
| Bi-Strategic | 0.31 | 0.25 | 0.36 | 0.70 |
| Prosocial | -0.64 | -0.67 | -0.60 | 0.40 |
| Antisocial | 1.32 | 1.23 | 1.41 | 1.07 |
| Rule Breaking | | | | |
| *Profile* | *M* | *LL* | *UL* | *SD* |
| Non-Strategic | -0.38 | -0.41 | -0.34 | 0.59 |
| Bi-Strategic | 0.11 | 0.06 | 0.17 | 0.67 |
| Prosocial | -0.67 | -0.70 | -0.64 | 0.41 |
| Antisocial | 1.40 | 1.30 | 1.49 | 1.08 |
| Affective Empathy | | | | |
| *Profile* | *M* | *LL* | *UL* | *SD* |
| Non-Strategic | -0.56 | -0.60 | -0.51 | 0.77 |
| Bi-Strategic | 0.66 | 0.61 | 0.72 | 0.71 |
| Prosocial | 0.69 | 0.64 | 0.75 | 0.73 |
| Antisocial | -0.55 | -0.63 | -0.47 | 0.97 |
| Cognitive Empathy | | | | |
| *Profile* | *M* | *LL* | *UL* | *SD* |
| Non-Strategic | -0.45 | -0.50 | -0.40 | 0.85 |
| Bi-Strategic | 0.38 | 0.32 | 0.44 | 0.75 |
| Prosocial | 0.77 | 0.73 | 0.82 | 0.60 |
| Antisocial | -0.51 | -0.60 | -0.41 | 1.14 |
