# Supplementary material for: The Upsides and Downsides of the Dark Side: A Longitudinal Study Into the Role of Prosocial and Antisocial Strategies in Close Friendship Formation
Source: Front Psychol. 2019 Feb 19;10:114. doi: 10.3389/fpsyg.2019.00114 (PMC6401596; doi:10.3389/fpsyg.2019.00114)
Supplement: Supplementary file 8 [file Table_8.docx]

# Table S8: Yearly Close friendship nominations and profile membership

|  |  | **Female Nominator** | | **Male Nominator** | |
| --- | --- | --- | --- | --- | --- |
| Profile | Nominated | Friends | SE | Friends | SE |
|  |  | **Grade 8** | | | |
| Non-Strategic | Male | 1.96 | 0.09 | 2.75 | 0.06 |
| Prosocial | Male | 3.03 | 0.11 | 3.15 | 0.08 |
| Bi-Strategic | Male | 3.99 | 0.12 | 2.91 | 0.1 |
| Antisocial | Male | 2.9 | 0.12 | 2.8 | 0.09 |
| Non-Strategic | Female | 2.94 | 0.1 | 1.58 | 0.11 |
| Prosocial | Female | 3.45 | 0.08 | 2.2 | 0.07 |
| Bi-Strategic | Female | 3.6 | 0.09 | 2.93 | 0.09 |
| Antisocial | Female | 2.95 | 0.13 | 2.18 | 0.15 |
|  |  | **Grade 9** | | | |
| Non-Strategic | Male | 2.29 | 0.07 | 3.08 | 0.05 |
| Prosocial | Male | 4 | 0.08 | 3.51 | 0.07 |
| Bi-Strategic | Male | 4.39 | 0.1 | 3.21 | 0.09 |
| Antisocial | Male | 3.11 | 0.09 | 3.03 | 0.08 |
| Non-Strategic | Female | 3.36 | 0.08 | 2.21 | 0.1 |
| Prosocial | Female | 3.85 | 0.06 | 2.66 | 0.06 |
| Bi-Strategic | Female | 3.63 | 0.07 | 2.92 | 0.08 |
| Antisocial | Female | 2.97 | 0.13 | 3.1 | 0.17 |
|  |  | **Grade 10** | | | |
| Non-Strategic | Male | 2.18 | 0.07 | 3.1 | 0.05 |
| Prosocial | Male | 3.82 | 0.08 | 3.85 | 0.07 |
| Bi-Strategic | Male | 3.94 | 0.1 | 3.01 | 0.09 |
| Antisocial | Male | 2.85 | 0.11 | 2.74 | 0.09 |
| Non-Strategic | Female | 3.33 | 0.07 | 2.29 | 0.1 |
| Prosocial | Female | 3.82 | 0.06 | 2.73 | 0.06 |
| Bi-Strategic | Female | 3.52 | 0.07 | 2.87 | 0.09 |
| Antisocial | Female | 3 | 0.13 | 2.9 | 0.18 |
|  |  | **Grade 11** | | | |
| Non-Strategic | Male | 2.62 | 0.06 | 2.79 | 0.06 |
| Prosocial | Male | 4.13 | 0.07 | 3.14 | 0.07 |
| Bi-Strategic | Male | 3.58 | 0.1 | 3.07 | 0.09 |
| Antisocial | Male | 2.26 | 0.12 | 2.83 | 0.11 |
| Non-Strategic | Female | 2.96 | 0.07 | 1.87 | 0.1 |
| Prosocial | Female | 3.83 | 0.05 | 2.68 | 0.06 |
| Bi-Strategic | Female | 3.54 | 0.06 | 2.06 | 0.1 |
| Antisocial | Female | 3.72 | 0.14 | 2.42 | 0.21 |
|  |  | **Grade 12** | | | |
| Non-Strategic | Male | 2.64 | 0.07 | 3.1 | 0.07 |
| Prosocial | Male | 3.81 | 0.09 | 3.22 | 0.08 |
| Bi-Strategic | Male | 3.62 | 0.11 | 2.99 | 0.11 |
| Antisocial | Male | 2.63 | 0.13 | 2.9 | 0.12 |
| Non-Strategic | Female | 3.18 | 0.08 | 2.08 | 0.11 |
| Prosocial | Female | 3.38 | 0.06 | 2.56 | 0.08 |
| Bi-Strategic | Female | 3.36 | 0.07 | 2.31 | 0.1 |
| Antisocial | Female | 2.2 | 0.18 | 2.07 | 0.24 |

Note: Opposite sex nominations shaded
